# First Aid Interventions for Intentional Caustic Chemical Assaults in Adults and Children: A Scoping Review

**DOI:** 10.7759/cureus.115776

**Published:** 2026-09-04

**Authors:** Jen Heng Pek, Nathan P Charlton, Phuong Thao Nguyen, Therese Djärv, Amy Kule

**Affiliations:** 1 Department of Emergency Medicine, Sengkang General Hospital, Singapore, SGP; 2 Emergency Medicine, University of Virginia Health System, Charlottesville, USA; 3 Department of Emergency Medicine, Medway Maritime Hospital, Gillingham, GBR; 4 Emergency Medicine, Karolinska Institute, Stockholm, SWE; 5 Department of Emergency Medicine, Loyola University Medical Center, Maywood, USA

**Keywords:** acid, alkali, caustic attack, eye, first aid, intervention, skin, treatment, vitriolage

## Abstract

There is limited evidence guiding optimal first aid management for caustic attacks, and this topic has not been systematically evaluated. We aimed to review the available literature to identify first aid treatments that demonstrate benefit. We searched MEDLINE, Embase, Cumulative Index to Nursing & Allied Health Literature, American Psychological Association PsycInfo, and Cochrane Library from inception through September 13, 2025. This scoping review was conducted in accordance with the methodological standards of the International Liaison Committee on Resuscitation Task Force Scoping Reviews, and reporting adhered to the Preferred Reporting Items for Systematic Reviews and Meta-analyses Extension for Scoping Reviews checklist. We identified six studies that met the inclusion criteria: two prospective cohort studies, three case reports, and one case series. An alkali was used in two studies, an acid in three studies, and an unknown caustic in one study. Three studies had victims with skin and eye involvement, one had a victim with only skin involvement, and two had victims with only eye involvement. The decontaminants included water, water and soap, physiological solution or normal saline, and Diphoterine®. The various outcomes reported for post-decontamination skin exposure included scarring, need for surgical intervention and grafting, re-epithelialization time, vision, and evisceration for eye exposure. Available evidence, despite being limited and of low certainty, consistently supports immediate removal of contaminated clothing and copious irrigation with water as a biologically plausible and clinically accepted first aid intervention.

## Introduction and background

The incidence of acid or alkali attacks, also referred to as caustic agent attacks or chemical assaults, is rising globally, with an estimated 10,000 cases annually worldwide [1]. The highest burden is borne by low- and middle-income countries, though incidence has increased in high-income regions as well [1]. Fatality is rare, but as the intention is to maim [2-4], survivors frequently experience lifelong disability, including scarring, visual impairment, repeated reconstructive surgeries, and are often accompanied by significant socioeconomic morbidity [4]. Caustic attacks can also have severe psychological sequelae, including anxiety, depression, and social isolation [5]. Family members, too, can be indirectly affected by the injury [5].

Epidemiologic data underscore the public health importance of these injuries. A review of data from India indicates an increasing burden of attacks from 2010 to 2019 [6], and data from Pakistan show approximately 200 cases of acid attacks annually [7]. In the United Kingdom, a 2012 study found that despite a decline in industrial chemical injuries over a 25-year period, there was a 42% increase in domestic chemical burns [8]. During an attack, caustic substances are often thrown into the victim’s face, causing permanent scarring and blindness. Commonly utilized agents are easily accessible through household and industrial cleaning products, including sodium hydroxide (NaOH), nitric acid (HNO_3_), sulfuric acid (H_2_SO_4_), and, in some regions, hydrofluoric acid (HF) [9-11]. Significant gaps remain in the evidence guiding optimal first aid management for these victims despite the introduction or proposal of regulatory measures by several countries [5].

The severity of injury following a caustic attack is determined by multiple factors, including the chemical’s concentration, volume, mechanism of tissue penetration, and duration of exposure [12]. Unlike thermal burns, caustic injuries may continue to progress until the agent is removed or neutralized, making early intervention critical. First aid measures, particularly rapid decontamination, represent an opportunity to meaningfully reduce tissue damage before advanced medical care is available [13,14]. A systematic review in 2022 identified several observational studies supporting early cool water lavage in improving clinical outcomes in chemical burns but concluded that current evidence was limited [13]. Therefore, it is intuitive that water lavage would play a major role in the treatment of caustic attacks. However, direct evidence guiding optimal first aid management of caustic attack remains sparse and largely extrapolated indirectly from industrial accidents and animal models, or from small observational studies.

Given the increasing global burden of caustic assaults and the central role of first aid in providing early care, the International Liaison Committee on Resuscitation (ILCOR) First Aid Task Force prioritized this topic for formal evaluation. This scoping review conducted by the First Aid Task Force of ILCOR, which provided the basis for the development of the Consensus on Science Treatment Recommendations (CoSTR), aims to systematically map the existing evidence on first aid interventions for caustic agent attacks, identify key outcomes and limitations in the literature, and highlight critical knowledge gaps to inform future research and guideline development.

## Review

Methodology

ILCOR uses a continuous evidence process to evaluate evidence and develop treatment recommendations for relevant first aid topics, culminating in the publication of a CoSTR. This scoping review was conducted in accordance with the methodological standards of ILCOR Task Force Scoping Reviews [15,16]. Reporting was done in accordance with the Preferred Reporting Items for Systematic Reviews and Meta-analyses Extension for Scoping Reviews (PRISMA-ScR) checklist [17], as provided in the Appendices. There is no published protocol or funding for this scoping review.

Selection Criteria

The published articles were screened and selected based on the following PICOST (Population, Intervention, Control, Outcomes, Study design, and Timeframe) [18], as shown in Table 1.

**Table 1:** Inclusion and exclusion criteria.

PICOST [18]	Description
Population	An adult or a child in an out-of-hospital setting subjected to a caustic agent attack to the skin or eye
Intervention	Intervention immediately available to the trained or untrained first aid provider (e.g., irrigation, cold compression, washing with soap, baking soda) and duration
Comparator	Other intervention and duration, or no intervention
Outcome	Clinical outcomes including pain relief, reduction in pain score, need for analgesia, extent of burn or tissue damage, intervention needed for burn or tissue damage, survival, adverse reaction from use of treatment (e.g., pain, erythema, allergy), and including harm to first aid providers
Study designs	Randomized controlled trials (RCTs), non-randomized studies (e.g., non-RCTs, interrupted time series, controlled before-and-after studies, cohort studies), case series and reports, conference proceedings and abstracts, as well as animal studies were included
Timeframe	Studies published in any year and in any language (if an English abstract was present) were eligible
Exclusion	Animal studies, review articles, commentaries, and editorials

Search Strategy

Search strings were developed by a medical librarian for the following databases: MEDLINE, Embase, Cumulative Index to Nursing & Allied Health Literature, American Psychological Association PsycInfo, and Cochrane Library. A search was performed from the inception date of the databases until September 13, 2025 (Appendices).

Study Selection

Records from the database searches were downloaded and imported into Covidence software for the removal of duplicates and screening [19]. Four authors (NPC, JHP, PN, AK) independently screened titles, abstracts, and full texts. Reasons for exclusion were documented, and any disagreements were discussed until a consensus was reached.

Data Collection

Four authors (NPC, JHP, PN, AK) participated in data extraction, with one author extracting the following data from each study: design, population, intervention(s), comparator and outcome(s), while another author verified the data for accuracy.

Data Synthesis

The results and findings of the included studies were presented as a descriptive narration.

Results

Literature Search and Study Selection

The search retrieved a total of 611 results from all databases, and after title and abstract screening, 21 studies were assessed for full-text eligibility. Six studies were ultimately selected for inclusion in the review [20-25]. Figure 1 illustrates the PRISMA diagram [19] for study selection.

**Figure 1:**
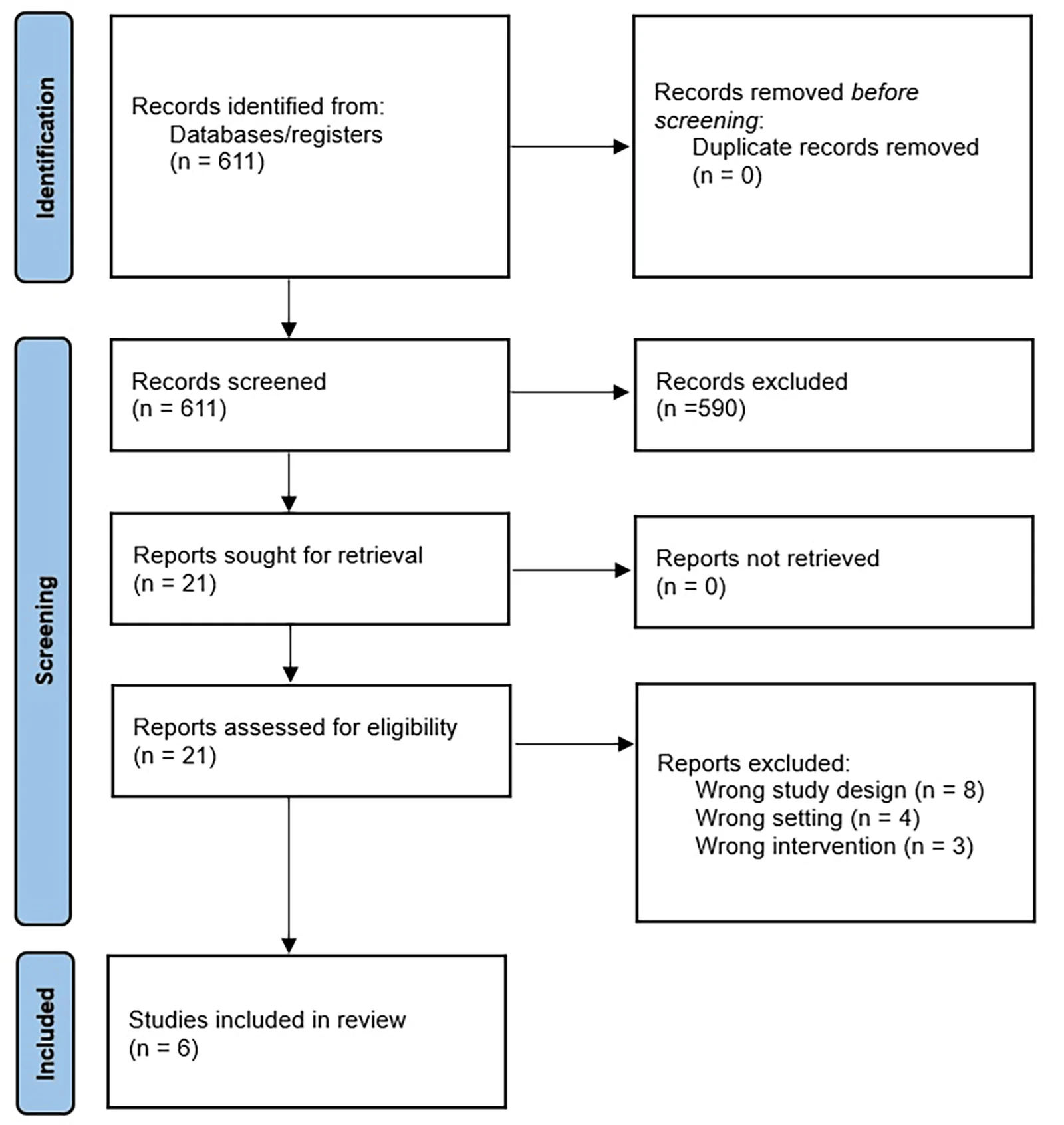
Preferred Reporting Items for Systematic Reviews and Meta-analyses (PRISMA) flow diagram for study selection. PRISMA diagram as created by Covidence software [19].

Study Characteristics

Among the six studies, there were two prospective cohort studies [21,24], one case series [22], and three case reports [20,23,25]. Regarding the location of the six studies, half (n = 3) occurred in Asia [21,23,25], whereas two were reported from North America [20,24] and one from Europe [22]. Considering the PROGRESS-Plus framework to describe equity factors [26,27], one case series [22] and one case report [20] involved males primarily, whereas in one case series [21] and two case reports [23,25] females were more commonly targeted. Age was frequently reported, with the majority affecting a younger population within the first four decades of life. Incidences occurred both in urban and rural settings, and an altercation was the underlying reason for the assault. Table 2 shows the study characteristics and findings.

**Table 2:** Study characteristics and findings of included studies.

Author, year	Country	Study design	Population	Intervention	Control	Study outcome and findings
D’Alessandro et al., 2020 [20]	United States	Case report	A 27-year-old man after assault with sulfuric acid	Soap and water, irrigation of corneas with Morgan’s lenses	None	Multiple surgeries, 102 days of hospital stay, 20/50 vision in the left eye, light perception in the right eye
Das et al., 2015 [21]	Bangladesh	Prospective cohort	126 patients with acid burns	Wash with plain water or normal saline, hydrocolloid dressing applied for 48–72 hours	None	Scarring, contractures, ophthalmic complications, deformities to the nose and ears
Kessel et al., 2015 [22]	Denmark	Case series	13 patients (19 eyes assaulted by sprayed ammonium hydroxide	Irrigation with saline, topical antibiotics and steroids, pupil dilation, sodium ascorbate (topical/systemic), and doxycycline (systemic). Necrotic tissue removal and symblepharon release; amniotic membrane transplantation or tarsorrhaphy for delayed healing	None	Severity at presentation by Roper Hall classification: 3 eyes grade 1–2; 10 eyes grade 3; 3 eyes grade 4. Vision outcomes: Of 7 eyes with ≤0.1 Snellen (20/200), 5 were eviscerated, 1 phthisic with light perception only, 1 recovered to 0.2 (40/200). Eye loss: 5 eyes enucleated, 1 phthisic (total 6/19 eyes, 31.6%). Mean time to removal was 9.4 months. Prognostic factors: poor initial visual acuity and full corneal epithelial defect
Leung et al., 2015 [23]	Hong Kong	Case report	One 16-year-old female victim of an acid assault – 8% total body surface area over face, scalp, back and upper limbs	Immediate water lavage, skin shaving thin layer removal, saline soaks, porcine skin test dressing, definitive split-thickness skin grafting	None	Complete healing with minimal hypertrophic scarring after early tangential shave; limited hypertrophic scarring in unshaved “acid run-off” tracks, corrected by minor revision. At the 1-year follow-up, excellent cosmetic and psychological recovery
Merle et al., 2005 [24]	French West Indies	Prospective cohort	66 patients (104 eyes) with alkali ocular burns – 30 (45.5%) due to assault	Initial irrigation with tap or mineral water carried out by victims (100%) 500 mL physiological solution – 48 eyes (46%)	500 mL Diphoterine – 56 eyes (54%)	Grade 1 and 2 burns had shorter time to reepithelization when rinsed with Diphoterine (1.9 ± 1 days) versus physiological solution (11.1 ± 1.4) days, and (5.6 ± 4.9) days and (10 ± 9.2) days, respectively
S.G.S., et al. 2025 [25]	Malaysia	Case report	A 21-year-old female assaulted with battery acid solution containing a high concentration of sulfuric acid during an altercation – 9% total body surface area over the face, chest, bilateral upper limbs, bilateral thighs and both eyes	Self: removed clothing above waist and rinsed with tap water. First responders: removed lower body clothing and irrigated	None	Upon arrival at the emergency department, eschar formation over the face, chest, bilateral upper limbs, bilateral thighs. Underwent early tangential excision, hydrosurgery, and skin allograft application over the chin, anterior chest, bilateral upper limbs, and bilateral thighs 3 days later. Underwent further debridement over face via hydrosurgical techniques and skin allograft application. Once allografts were rejected, 2 weeks later, another wound debridement and thick split-thickness skin graft was applied over her face, neck and bilateral thighs

A total of 208 patients were identified across the six studies. In two studies, the caustic agent was identified as an alkali [22,24], an acid in three studies [20,21,25], and unknown in one study [23]. Three studies had victims with skin and eye involvement [20,21,25], one had a victim with only skin involvement [23], and two had victims with only eye involvement [22,24]. The agents used for decontamination included water [21,23-25], water and soap [20], physiological solution or normal saline [21,22,24], and Diphoterine® [24]. Pre-hospital decontamination was performed as first aid in three studies [21,24,25], and in the emergency department or hospital in three studies [20,22,23].

Outcomes

Reported outcomes included scarring [21,23], need for surgical [20,21,23,25] and grafting [21,23,25] for skin exposure and eye evisceration [22], time to re-epithelialization [24], and vision [20,22] for eye exposure.

Das et al. provided observational evidence in a prospective cohort that first aid water irrigation reduces burn depth and subsequent scarring in victims of acid assault [21]. In their study, 93 of the 126 patients with acid burns who received immediate water irrigation had significantly lower rates of hypertrophic scarring (11.4% vs. 48.5%) with a relative risk of 0.24 (95% confidence interval = 0.13-0.47). Patients presenting with superficial or superficial partial-thickness injuries were noted to receive water irrigation, requiring fewer surgical interventions. A case series by Kessel et al. (2015) described severe ammonium hydroxide ocular injuries and initial irrigation with saline among 13 patients [22]. They did not evaluate the efficacy of saline relative to other treatments, and outcomes appeared primarily driven by initial injury severity, with many eyes progressing to phthisis or evisceration. Irrigation was performed either as a first aid measure or initial hospital treatment in several case reports and case series [20,23,25]; however, we were unable to draw any conclusions from these articles due to the nature of these reports [18].

One prospective cohort study evaluated Diphoterine®, a commercially available amphoteric irrigation solution for the skin and eyes, compared to physiologic saline for ocular alkali burns [24]. Time to corneal re-epithelialization was substantially shorter with Diphoterine® irrigation among grade 1-2 injuries. Data on severe (grade 3-4) burns were insufficient for meaningful conclusions. [1] Similar to the skin studies, delays to irrigation correlated with injury severity, which reinforces the importance of early decontamination.

Discussion

This scoping review identified a limited body of evidence addressing first aid interventions for caustic attacks. Across six included studies, comprising prospective cohorts, case series, and case reports, there was consistent but low-certainty evidence supporting immediate and copious irrigation as an early intervention for both skin and ocular exposures. No randomized controlled trials were identified, reflecting the challenges inherent in studying intentional chemical injuries.

For chemical burns, the goal of treatment is to limit exposure and long-term impairment [3,11]. Current recommendations for first aid following accidental chemical exposure consistently emphasize that the first step should be immediate removal of contaminated clothing, followed by copious irrigation with water [28,29]. Water acts through several mechanisms: physically removing the chemical from the skin or eye, diluting the offending agent, and restoring tissue hydration and pH [12,30]. Although treatments could be intuitively extrapolated from industrial accidental chemical injury, the pattern of exposure and injury may not necessarily be the same [8,13,21]. For instance, there is a preponderance of caustic attacks directed at the face compared with other areas of the body, and due to the deliberate nature of the attack, there is a potential for larger volumes and stronger types of caustic agents which differ from industrial and accidental injury [21]. Important knowledge gaps persist regarding how lay providers can best deliver first aid for caustic attacks, including optimal irrigation parameters and the role of specialized solutions. As no prior review has systematically mapped the direct evidence addressing first aid interventions for intentional caustic attacks in adults and children, this scoping review adds important information to the current medical literature that begins to address these gaps.

The strongest direct human evidence for the treatment of caustic chemical attack comes from observational cohort data. In victims of acid assault, prompt water irrigation was associated with a markedly lower risk of hypertrophic scarring (relative risk = 0.2440; 95% confidence interval = 0.1265 -0.4706) and reduced need for surgical intervention [21]. These findings are clinically significant, given that scarring and reconstructive surgery represent major drivers of long-term disability and healthcare utilization among survivors. Early self-initiated or hospital-initiated irrigation was documented in multiple case reports and case series; however, while supporting the concept of early irrigation, conclusions could not be drawn from this data due to a lack of controls [20,22,23,25]. While conclusions cannot be drawn due to the observational nature of this data, one animal study suggested that the protective effect of irrigation is most pronounced when initiated early, reinforcing the concept that contact time is a critical determinant of tissue damage [31].

Evidence supporting the use of specialized decontamination agents, including amphoteric solutions, remains limited. A single prospective cohort study reported more rapid corneal re-epithelialization with Diphoterine® compared with saline in patients with mild-to-moderate alkali eye injuries, but data were insufficient to draw conclusions for severe burns or acid exposures [24]. Further limiting utility, these products are not widely available in many regions, and recommendations that depend on specialized agents may widen existing disparities in first aid care. In contrast, clean water is more broadly accessible, inexpensive, and familiar to lay responders, making it the most practical first aid option in most regions of the world.

Limitations

This review also highlights several important gaps in the literature. Few studies specifically evaluate first aid delivered in the prehospital or lay rescuer setting, despite the concept that immediate intervention is the most impactful intervention. No studies directly compared different irrigation volumes or durations, evaluated the role of clothing removal, and only one study evaluated an alternative decontamination approach in situations where water is not readily available. In addition, reported outcomes varied, further limiting meaningful comparison across studies.

Because randomization of immediate intervention techniques is often difficult and potentially unethical, research in this area will often rely on observational designs, registries, and animal models. Public health interventions, such as community education campaigns, may provide valuable opportunities to assess the effectiveness of first aid indirectly by examining changes in injury severity, surgical requirements, and long-term outcomes over time.

## Conclusions

Caustic attacks are an increasing global problem with the potential for profound physical and psychosocial consequences. Current low-certainty evidence and mechanistic rationale support immediate removal of contaminated clothing and copious water irrigation as the most practical first aid intervention following caustic assault exposure. These interventions are associated with reduced tissue injury and improved clinical outcomes. Future research should focus on optimizing delivery of irrigation, including duration and volume, improving public education, and evaluating alternative strategies to ensure timely decontamination across diverse resource settings.

## Figures and Tables

**Figure 2:**
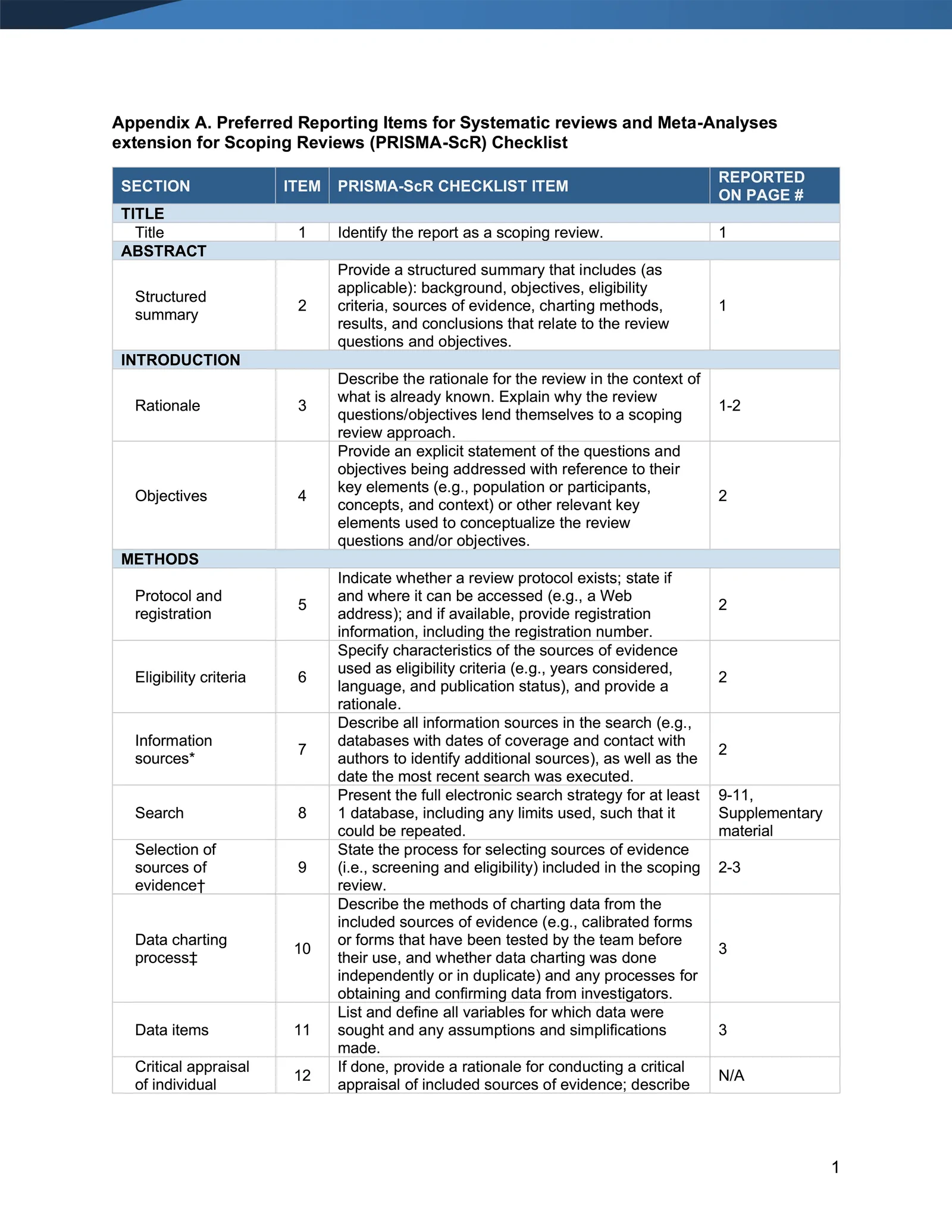
Preferred Reporting Items for Systematic Reviews and Meta-Analyses Extension for Scoping Reviews (PRISMA-ScR) checklist (page 1). [17].

**Figure 3:**
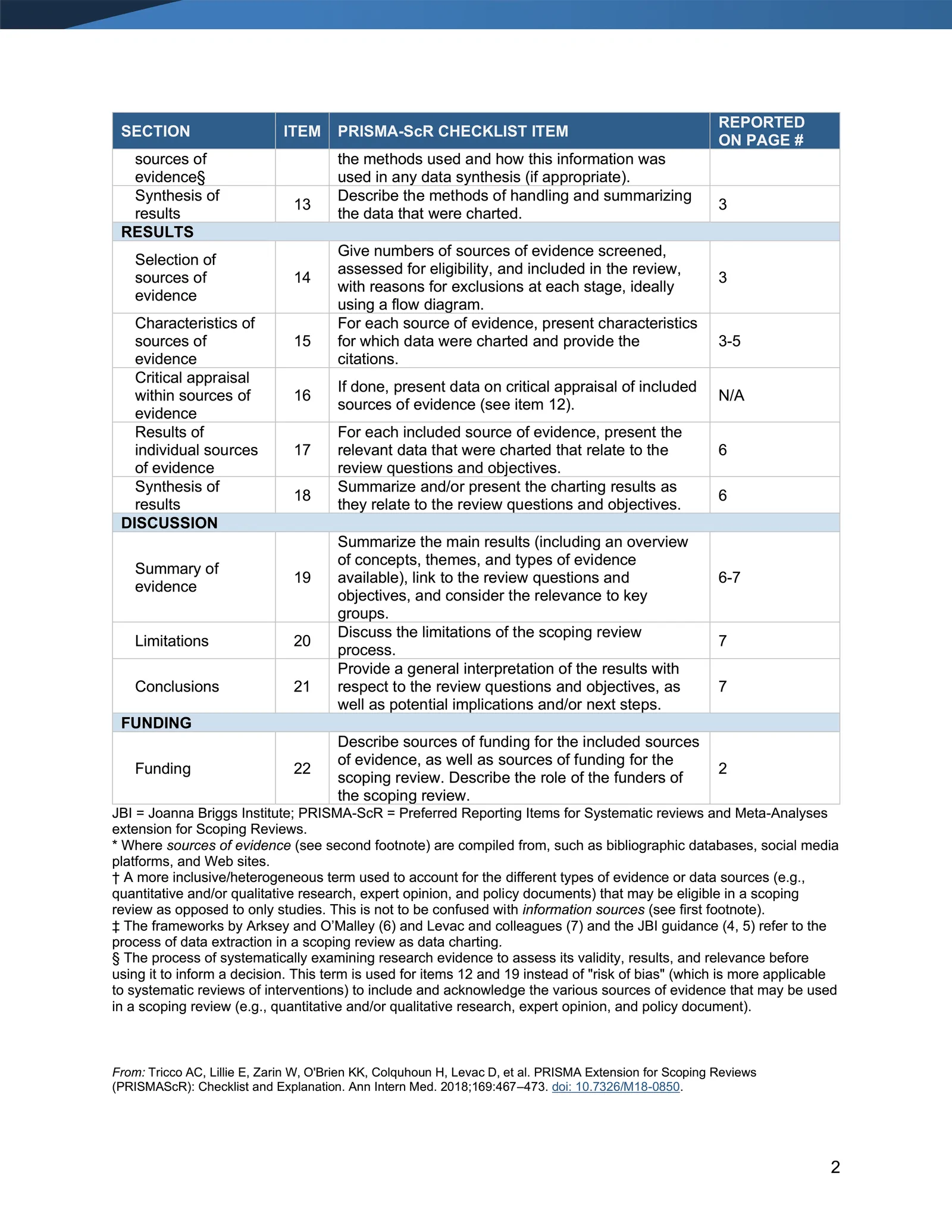
Preferred Reporting Items for Systematic Reviews and Meta-Analyses Extension for Scoping Reviews (PRISMA-ScR) checklist (page 2).

## Appendices

**Table 3:** Search strategy details (September 13, 2025).

#	Concept	Keywords	MeSH
1	Acid attack	“acid attack”[tiab:~3] OR “acid attacks”[tiab:~3] OR “acid throw”[tiab:~3] OR “acid throwing”[tiab:~3] OR “acid assault”[tiab:~3] OR “vitriol attack”[tiab:~3] OR “vitriol attacks”[tiab:~3] OR “vitriol throw”[tiab:~3] OR “vitriol throwing”[tiab:~3] OR vitriolage[tiab] OR vitriolism[tiab] OR “chemical assault”[tiab:~3] OR “assault corrosive”[tiab:~3] OR “violence corrosive”[tiab:~3] OR “throw corrosive”[tiab:~3] OR “corrosive attack”[tiab:~3] OR “corrosive attacks”[tiab:~3]	
2	First aid setting	“emergenc*”[tiab] OR prehospital[tiab] OR “pre-hospital”[tiab] OR paramedic*[tiab] OR “rescue personnel”[tiab] OR “emergency respon*”[tiab] OR EMS[tiab] OR “emergency medical technician*”[tiab] OR EMT[tiab] OR “first respon*”[tiab] OR bystander*[tiab] OR “first-line”[tiab] OR “lay rescue*”[tiab] OR “lay person*”[tiab] OR layperson*[tiab] OR “lay population”[tiab] OR emergen*[tiab] OR immediat*[tiab]	“Emergency Service, Hospital”[MeSH] OR “Emergency Medical Services”[MeSH] OR “First Aid”[MeSH]
3	Acid and alkali substances and burns	Alkali*[tiab] OR “sodium hydroxide”[tiab] OR “potassium hydroxide”[tiab] OR caustic*[tiab] OR acid[tiab] OR chemical*[tiab] OR burn*[tiab] OR “corrosive substance*”[tiab]	Alkalies[MeSH] OR Acids[MeSH] OR Caustics[MeSH] OR “Burns, Chemical”[MeSH] OR “Burns, Inhalation”[MeSH] OR “Eye Burns”[MeSH]
4	Treatment	treat*[tiab] OR therap*[tiab] OR care[tiab] OR “first aid”[tiab] OR “initial assist*”[tiab] OR “first respon*”[tiab] OR “first assist*”[tiab]	Therapy[sh]
	Limits	NOT (“Animals”[Mesh] NOT (“Animals”[Mesh] AND “Humans”[Mesh])) AND english[la] NOT (comment[pt] OR editorial[pt] OR news[pt])	

**Table 4:** Search query and results from Cochrane.

Cochrane Library via Wiley, Search Manager	
Strategy 1	((acid NEAR/4 attack):ti,ab,kw OR (acid NEAR/4 attacks):ti,ab,kw OR (acid NEAR/4 throw):ti,ab,kw OR (acid NEAR/4 throwing):ti,ab,kw OR (acid NEAR/4 assault):ti,ab,kw OR (vitriol NEAR/4 attack):ti,ab,kw OR (vitriol NEAR/4 attacks):ti,ab,kw OR (vitriol NEAR/4 throw):ti,ab,kw OR (vitriol NEAR/4 throwing):ti,ab,kw OR vitriolage:ti,ab,kw OR vitriolism:ti,ab,kw OR (chemical NEAR/4 assault):ti,ab,kw OR (assault NEAR/4 corrosive):ti,ab,kw OR (violence NEAR/4 corrosive):ti,ab,kw OR (throw NEAR/4 corrosive):ti,ab,kw OR (corrosive NEAR/4 attack):ti,ab,kw OR (corrosive NEAR/4 attacks):ti,ab,kw) AND (emergenc*:ti,ab,kw OR prehospital:ti,ab,kw OR pre-hospital:ti,ab,kw OR paramedic*:ti,ab,kw OR rescue personnel:ti,ab,kw OR emergency respon*:ti,ab,kw OR ems:ti,ab,kw OR emergency medical technician*:ti,ab,kw OR emt:ti,ab,kw OR bystander*:ti,ab,kw OR first-line:ti,ab,kw OR lay rescue*:ti,ab,kw OR lay person*:ti,ab,kw OR layperson*:ti,ab,kw OR lay population:ti,ab,kw OR emergen*:ti,ab,kw OR immediat*:ti,ab,kw OR [mh “Emergency Service, Hospital”] OR [mh “Emergency Medical Services”] OR [mh “First Aid”] OR treat*:ti,ab,kw OR therap*:ti,ab,kw OR care:ti,ab,kw OR first aid:ti,ab,kw OR initial assist*:ti,ab,kw OR first respon*:ti,ab,kw OR first assist*:ti,ab,kw)
Strategy 2	(alkali*:ti,ab,kw OR “sodium hydroxide”:ti,ab,kw OR “potassium hydroxide”:ti,ab,kw OR “sulfuric acid”:ti,ab,kw OR “nitric acid”:ti,ab,kw OR “hydrochloric acid”:ti,ab,kw OR “hydrofluoric acid”:ti,ab,kw OR “acid attack”:ti,ab,kw OR caustic:ti,ab,kw OR “chemical burn”:ti,ab,kw OR “corrosive substance”:ti,ab,kw OR [mh Caustics] OR [mh “Burns, Chemical”] OR [mh “Burns, Inhalation”] OR [mh “Eye Burns”]) AND (treat*:ti,ab,kw OR therap*:ti,ab,kw OR care:ti,ab,kw OR “first aid”:ti,ab,kw OR assist*:ti,ab,kw OR first respon*:ti,ab,kw) AND (vitriol*:ti,ab,kw OR violen*:ti,ab,kw OR terroris*:ti,ab,kw OR [mh terrorism] OR [mh violence])

**Table 5:** Search query and results from Medline database.

MEDLINE via PubMed	
Strategy 1: 1 AND (2 OR 3) (first aid/therapy specific to acid attacks) n = 341 on 9/13/25	(“acid attack”[tiab:~3] OR “acid attacks”[tiab:~3] OR “acid throw”[tiab:~3] OR “acid throwing”[tiab:~3] OR “acid assault”[tiab:~3] OR “vitriol attack”[tiab:~3] OR “vitriol attacks”[tiab:~3] OR “vitriol throw”[tiab:~3] OR “vitriol throwing”[tiab:~3] OR vitriolage[tiab] OR vitriolism[tiab] OR “chemical assault”[tiab:~3] OR “assault corrosive”[tiab:~3] OR “violence corrosive”[tiab:~3] OR “throw corrosive”[tiab:~3] OR “corrosive attack”[tiab:~3] OR “corrosive attacks”[tiab:~3]) AND (“emergenc*”[tiab] OR prehospital[tiab] OR “pre-hospital”[tiab] OR paramedic*[tiab] OR “rescue personnel”[tiab] OR “emergency respon*”[tiab] OR EMS[tiab] OR “emergency medical technician*”[tiab] OR EMT[tiab] OR “first respon*”[tiab] OR bystander*[tiab] OR “first-line”[tiab] OR “lay rescue*”[tiab] OR “lay person*”[tiab] OR layperson*[tiab] OR “lay population”[tiab] OR emergen*[tiab] OR immediat*[tiab] OR “Emergency Service, Hospital”[MeSH] OR “Emergency Medical Services”[MeSH] OR “First Aid”[MeSH] OR treat*[tiab] OR therap*[tiab] OR care[tiab] OR “first aid”[tiab] OR “initial assist*”[tiab] OR “first respon*”[tiab] OR “first assist*”[tiab] OR Therapy[sh]) NOT (“Animals”[Mesh] NOT (“Animals”[Mesh] AND “Humans”[Mesh])) AND english[la] NOT (comment[pt] OR editorial[pt] OR news[pt])
Strategy 2: 3 AND 4 AND (assault OR attack OR vitriol OR Violence[MeSH]) (therapy/treatment for acid substances used in attacks) n = 114 on 9/13/25	(alkali*[tiab] OR “sodium hydroxide”[tiab] OR “potassium hydroxide”[tiab] OR “sulfuric acid*”[tiab] OR “nitric acid”[tiab] OR “hydrochloric acid”[tiab] OR “hydrofluoric acid”[tiab] OR “acid attack*”[tiab] OR caustic*[tiab] OR “chemical burn*”[tiab] OR “corrosive substance*”[tiab] OR Caustics[MeSH] OR “Burns, Chemical”[MeSH] OR “Burns, Inhalation”[MeSH] OR “Eye Burns”[MeSH]) AND (treat*[tiab] OR therap*[tiab] OR care[tiab] OR “first aid”[tiab] OR assist*[tiab] OR “first respon*”[tiab] OR Therapy[sh]) AND (vitriol*[tiab] OR violen*[tiab] OR terroris*[tiab] OR Violence[MeSH]) NOT (“Animals”[Mesh] NOT (“Animals”[Mesh] AND “Humans”[Mesh])) AND english[la] NOT (comment[pt] OR editorial[pt] OR news[pt])

**Table 6:** Search query and results from Embase.

Embase via Embase.com (Elsevier)	
Strategy 1: 1 AND (2 OR 3) (first aid/therapy specific to acid attacks) n = 280 on 9/13/25	(((‘acid’ NEAR/4 ‘attack’):ti,ab,kw) OR ((‘acid’ NEAR/4 ‘attacks’):ti,ab,kw) OR ((‘acid’ NEAR/4 ‘throw’):ti,ab,kw) OR ((‘acid’ NEAR/4 ‘throwing’):ti,ab,kw) OR ((‘acid’ NEAR/4 ‘assault’):ti,ab,kw) OR ((‘vitriol’ NEAR/4 ‘attack’):ti,ab,kw) OR ((‘vitriol’ NEAR/4 ‘attacks’):ti,ab,kw) OR ((‘vitriol’ NEAR/4 ‘throw’):ti,ab,kw) OR ((‘vitriol’ NEAR/4 ‘throwing’):ti,ab,kw) OR ‘vitriolage’:ti,ab,kw OR ‘vitriolism’:ti,ab,kw OR ((‘chemical’ NEAR/4 ‘assault’):ti,ab,kw) OR ((‘assault’ NEAR/4 ‘corrosive’):ti,ab,kw) OR ((‘violence’ NEAR/4 ‘corrosive’):ti,ab,kw) OR ((‘throw’ NEAR/4 ‘corrosive’):ti,ab,kw) OR ((‘corrosive’ NEAR/4 ‘attack’):ti,ab,kw) OR ((‘corrosive’ NEAR/4 ‘attacks’):ti,ab,kw) OR ‘acid attack (violence)’/exp) AND (‘emergenc*’:ti,ab,kw OR ‘prehospital’:ti,ab,kw OR ‘pre-hospital’:ti,ab,kw OR ‘paramedic*’:ti,ab,kw OR ‘rescue personnel’:ti,ab,kw OR ‘emergency respon*’:ti,ab,kw OR ‘ems’:ti,ab,kw OR ‘emergency medical technician*’:ti,ab,kw OR ‘emt’:ti,ab,kw OR ‘bystander*’:ti,ab,kw OR ‘first-line’:ti,ab,kw OR ‘lay rescue*’:ti,ab,kw OR ‘lay person*’:ti,ab,kw OR ‘layperson*’:ti,ab,kw OR ‘lay population’:ti,ab,kw OR ‘emergen*’:ti,ab,kw OR ‘immediat*’:ti,ab,kw OR ‘first responder (person)’/exp OR ‘emergency care’/exp OR ‘first aid’/de OR ‘emergency treatment’/de OR ‘rescue personnel’/de OR ‘emergency health service’/exp/mj OR ‘treat*’:ti,ab,kw OR ‘therap*’:ti,ab,kw OR ‘care’:ti,ab,kw OR ‘first aid’:ti,ab,kw OR ‘initial assist*’:ti,ab,kw OR ‘first respon*’:ti,ab,kw OR ‘first assist*’:ti,ab,kw) AND [english]/lim NOT ([animals]/lim NOT [humans]/lim) NOT (‘conference abstract’/it OR ‘editorial’/it OR ‘clinical trial’/it OR ‘comment’/it OR ‘letter’/it)
Strategy 2: 3 AND 4 AND (assault OR attack OR vitriol OR Violence) (therapy/treatment for acid substances used in attacks) n = 141 on 9/13/25	(‘alkali*’:ti,ab,kw OR ‘sodium hydroxide’:ti,ab,kw OR ‘potassium hydroxide’:ti,ab,kw OR ‘sulfuric acid*’:ti,ab,kw OR ‘nitric acid’:ti,ab,kw OR ‘hydrochloric acid’:ti,ab,kw OR ‘hydrofluoric acid’:ti,ab,kw OR ‘acid attack*’:ti,ab,kw OR ‘caustic*’:ti,ab,kw OR ‘chemical burn*’:ti,ab,kw OR ‘corrosive substance*’:ti,ab,kw OR ‘acid attack (violence)’/exp OR ‘caustic agent’/exp/mj OR ‘chemical burn’/exp/mj OR ‘lung burn’/exp/mj OR ‘eye burn’/exp/mj) AND (‘treat*’:ti,ab,kw OR ‘therap*’:ti,ab,kw OR ‘care’:ti,ab,kw OR ‘first aid’:ti,ab,kw OR ‘first aid’/de OR ‘assist*’:ti,ab,kw OR ‘first respon*’:ti,ab,kw) AND (‘vitriol*’:ti,ab,kw OR ‘violen*’:ti,ab,kw OR ‘terroris*’:ti,ab,kw OR ‘terrorism’/exp/mj OR ‘violence’/exp/mj) AND [english]/lim NOT ([animals]/lim NOT [humans]/lim) NOT (‘conference abstract’/it OR ‘editorial’/it OR ‘clinical trial’/it OR ‘comment’/it OR ‘letter’/it)

**Table 7:** Search query and results from Cumulative Index to Nursing & Allied Health Literature (CINAHL).

Cumulative Index to Nursing & Allied Health Literature with Full Text via EBSCOHost
(XB (acid N3 attack) OR (acid N3 attacks) OR (acid N3 throw) OR (acid N3 throwing) OR (acid N3 assault) OR (vitriol N3 attack) OR (vitriol N3 attacks) OR (vitriol N3 throw) OR (vitriol N3 throwing) OR vitriolage OR vitriolism OR (chemical N3 assault) OR (assault N3 corrosive) OR (violence N3 corrosive) OR (throw N3 corrosive) OR (corrosive N3 attack) OR (corrosive N3 attacks)) AND XB (emergenc* OR prehospital OR pre-hospital OR paramedic* OR “rescue personnel” OR “emergency respon*” OR ems OR “emergency medical technician*” OR emt OR bystander* OR first-line OR “lay rescue*” OR “lay person*” OR layperson* OR “lay population” OR emergen* OR immediat* OR treat* OR therap* OR care OR “first aid” OR “initial assist*” OR “first respon*” OR “first assist*” OR (MH "Emergency Responders+") OR (MH "Prehospital Care") OR (MH "Red Cross") OR (MH "American Red Cross") OR (MH "Emergency Treatment") OR (MH "First Aid") OR (MH “Emergency Medical Services”))) OR (XB (“alkali*” OR “sodium hydroxide” OR “potassium hydroxide” OR “sulfuric acid*” OR “nitric acid” OR “hydrochloric acid” OR “hydrofluoric acid” OR “acid attack*” OR “caustic*” OR “chemical burn*” OR “corrosive substance*”) AND XB (treat* OR therap* OR care OR “first aid” OR “initial assist*” OR “first respon*” OR “first assist*”) AND (XB (vitriol* OR violen* OR terroris*) OR (MH Violence) OR (MH Terrorism))

**Table 8:** Search query and results from American Psychological Association PsycInfo.

American Psychological Association PsycInfo via EBSCOHost
(XB (acid N3 attack) OR (acid N3 attacks) OR (acid N3 throw) OR (acid N3 throwing) OR (acid N3 assault) OR (vitriol N3 attack) OR (vitriol N3 attacks) OR (vitriol N3 throw) OR (vitriol N3 throwing) OR vitriolage OR vitriolism OR (chemical N3 assault) OR (assault N3 corrosive) OR (violence N3 corrosive) OR (throw N3 corrosive) OR (corrosive N3 attack) OR (corrosive N3 attacks)) AND XB (emergenc* OR prehospital OR pre-hospital OR paramedic* OR “rescue personnel” OR “emergency respon*” OR ems OR “emergency medical technician*” OR emt OR bystander* OR first-line OR “lay rescue*” OR “lay person*” OR layperson* OR “lay population” OR emergen* OR immediat* OR treat* OR therap* OR care OR “first aid” OR “initial assist*” OR “first respon*” OR “first assist*”)) OR (XB (“alkali*” OR “potassium hydroxide” OR “sulfuric acid*” OR “nitric acid” OR “sodium hydroxide” OR “hydrochloric acid” OR “hydrofluoric acid” OR “acid attack*” OR “caustic*” OR “chemical burn*” OR “corrosive substance*”) AND XB (treat* OR therap* OR care OR “first aid” OR “initial assist*” OR “first respon*” OR “first assist*”) AND (XB (vitriol* OR violen* OR terroris*) OR DE "Violence" OR DE "Terrorism"))
